# Supplementary material for: Differential SNARE chaperoning by Munc13-1 and Munc18-1 dictates fusion pore fate at the release site
Source: Nat Commun. 2024 May 16;15:4132. doi: 10.1038/s41467-024-46965-7 (PMC11099066; doi:10.1038/s41467-024-46965-7)
Supplement: Supplementary file 1 — Supplementary Information [file 41467_2024_46965_MOESM1_ESM.pdf]

Supplementary Information

Differential SNARE chaperoning by Munc13-1 and Munc18-1 dictates fusion pore fate at the release site

Bhavya R. Bhaskar[1], Laxmi Yadav[1], Malavika Sriram[1], Kinjal Sanghrajka[1], Mayank Gupta[1], Boby K. V.[1], Rohith K. Nellikka[1], Debasis Das[1]†

[1]Department of Biological Sciences, Tata Institute of Fundamental Research, Mumbai 400005, India.

†Corresponding author: E-mail: debasis.das@tifr.res.in

Fig. S1

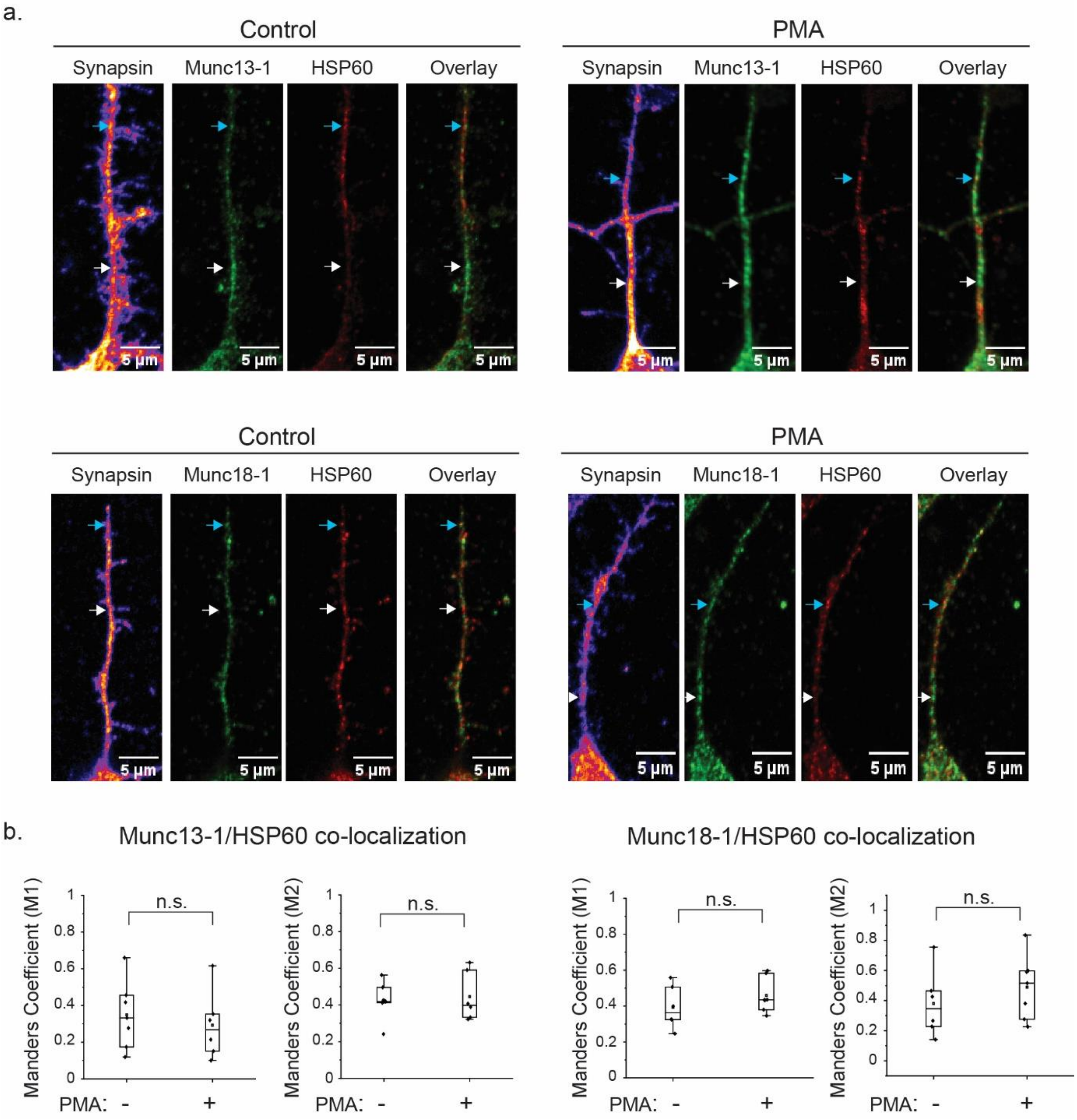

Fig. S1: Co-localization of Hsp60 with Munc13-1 and Munc18-1 in rat cortical neurons.

a, Representative overlay confocal image of neurites that were triple labeled for synapsin (Alexa 647-conjugated secondary antibody), Hsp60 (Alexa 555-conjugated secondary antibody), and the SNARE chaperone Munc13-1 (upper panel), Munc18-1 (lower panel) (Alexa 488-conjugated secondary antibody), in the absence (left) and presence (right) of the phorbol ester PMA. Blue arrows: co-localization; while arrows: no co-localization. **b**, The scatter plots showing Manders correlation coefficients M1 and M2 for Hsp60's co-localization with Munc13-1 (left) and Munc18-1 (right), under indicated conditions. The box plot minima and maxima represent the 25th and 75th percentiles, the lower and upper whiskers indicate

the 5th and 95th percentiles and the center line and square indicate median and mean respectively; n=6 (Munc13-1/HSP60 and Munc18-1/HSP60) individual neurons. Student's T test (one tailed) was performed to compare the two means; n.s. - non-significance.

Fig. S2

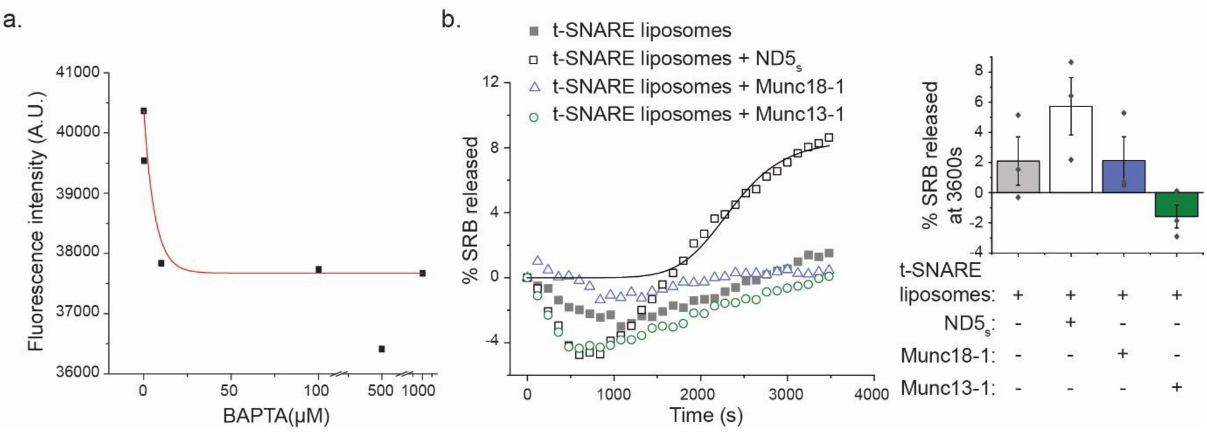

Fig. S2: BAPTA titration and leakage assay.

a, Representative plot shows fluorescence intensity of calcium sensor Fluo-4 for different [BAPTA], as indicated. **b,** Representative plot for leakage assay shows percent SRB released from t-SNARE liposomes in the absence and presence of SNARE chaperones and during membrane fusion in the presence of $ND5_S$. Inset (right) bar plot indicating mean±SEM indicating average percent of SRB released at 3600s for different conditions; n = 3 independent trials.

Fig. S3

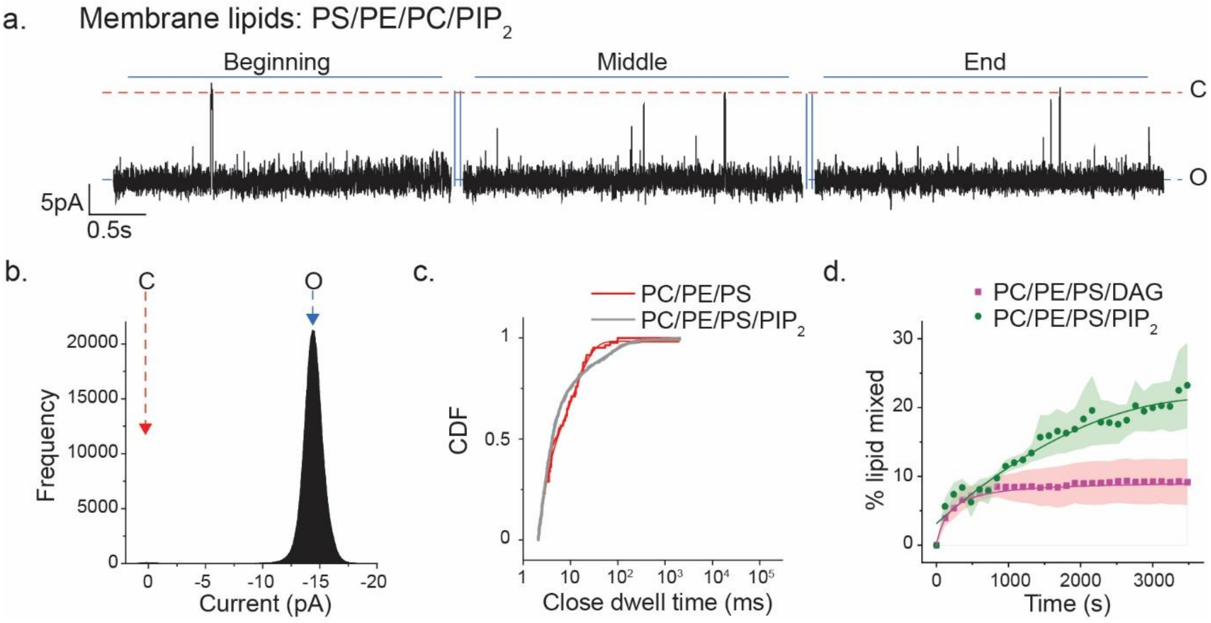

Fig. S3: Effect of PIP_2 on $ND5_S$ pore.

a, Representative trace of single $ND5_S$ pore formed. Three epochs (beginning, middle and end) of the trace are shown. Membrane lipid composition of the BLMs is highlighted above the trace. Full closed (C) and full open (O) states of the individual pores are indicated with the respective currents. **b,** Current histogram of the pore from **a**. Closed (C) and full open (O) states are indicated by red and blue arrows. **c,** Cumulative distribution functions (CDF) of closed dwell times for each experimental condition. n = 3 independent BLMs; three sets of NDs were used for each of the conditions. **d,** Scatter plot showing time course of averaged percentage lipid mixed during fusion reaction between $ND5_S$ and t-SNARE liposomes containing PIP_2 and DAG lipids as indicated. Light colour area under the curve indicates SEM for each time point. n=4 independent trials were done for each condition.

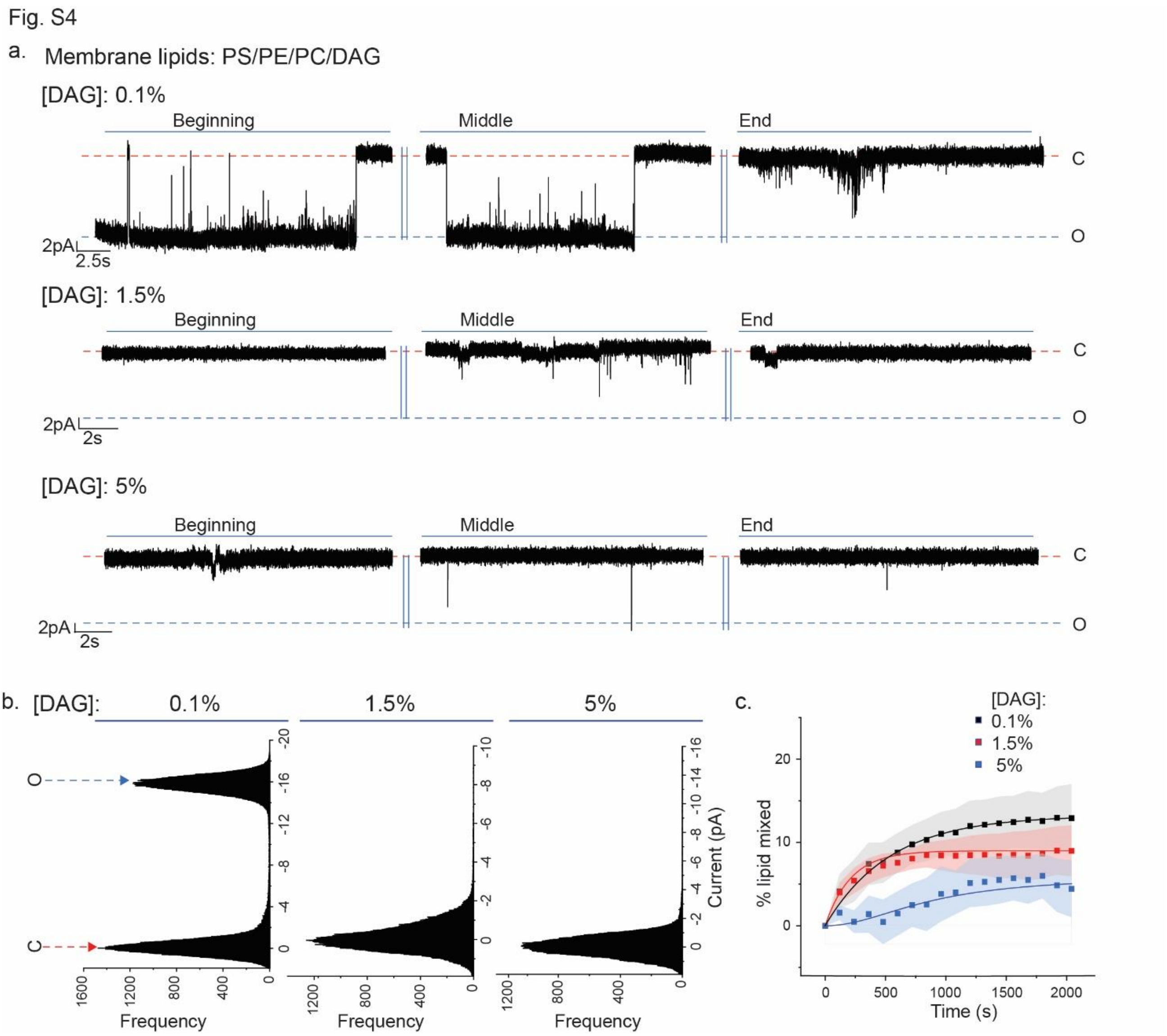

Fig. S4: Effect of DAG titration on membrane fusion.

a,b, Representative raw traces of ND5$_S$ pores (a) and the corresponding current histograms (b) are shown, for different [DAG] in the BLM, as indicated in the figure. n = 3 independent BLMs; two sets of NDs were used for each of the conditions. **c,** Scatter plot showing time course of averaged percentage lipid mixed during fusion reaction between ND5$_S$ and t-SNARE liposomes containing PE/PS/PC and various [DAG] lipids as indicated. Light colour area under the curve indicates SEM for each time point. n=4 independent trials were done for each condition.

Fig. S5

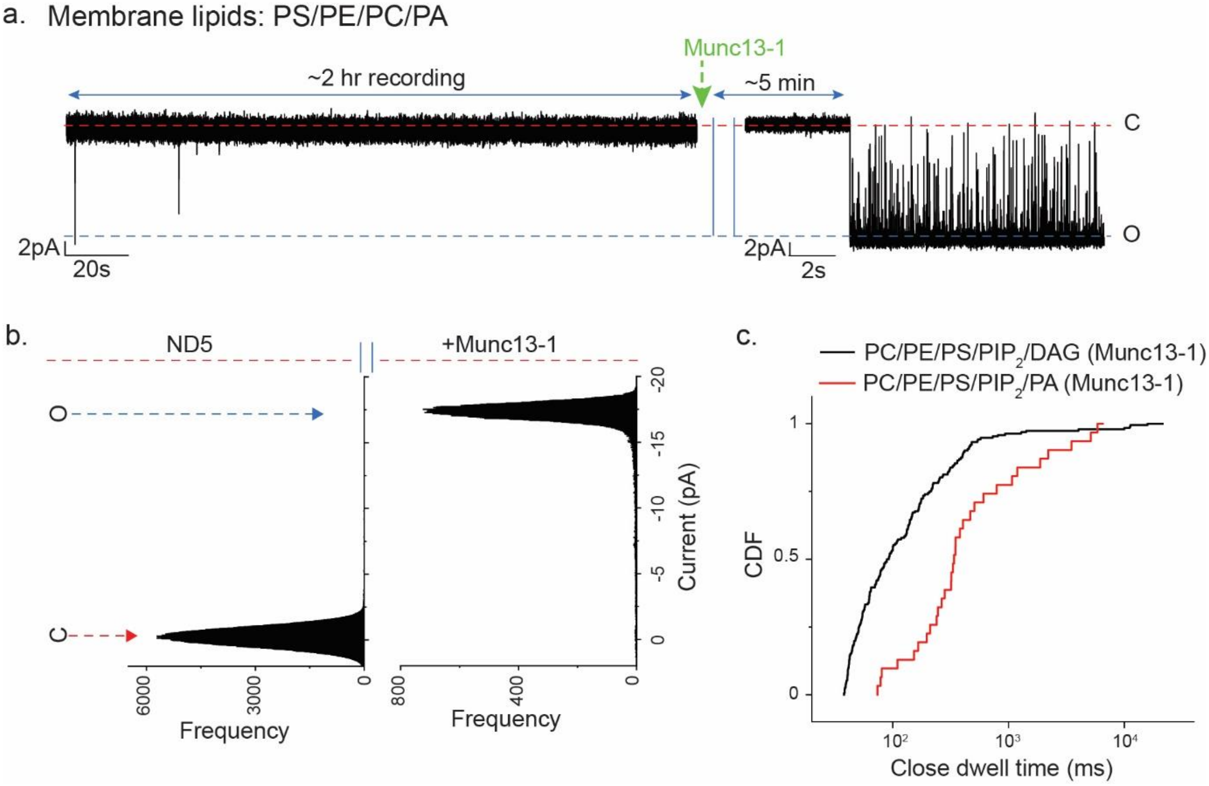

Fig. S5: Effect of Phosphatidic acid (PA) in ND5$_S$ pores.

a,b, Representative trace (a) and the corresponding current histograms (b) of ND5$_S$ pore, before and after Munc13-1 addition. The BLM lipid composition is mentioned. The close (C) and open (O) states are indicated. n = 3 independent BLM recordings. **c,** Cumulative distribution functions (CDF) of closed dwell times for each experimental condition as indicated.

Fig. S6

a.

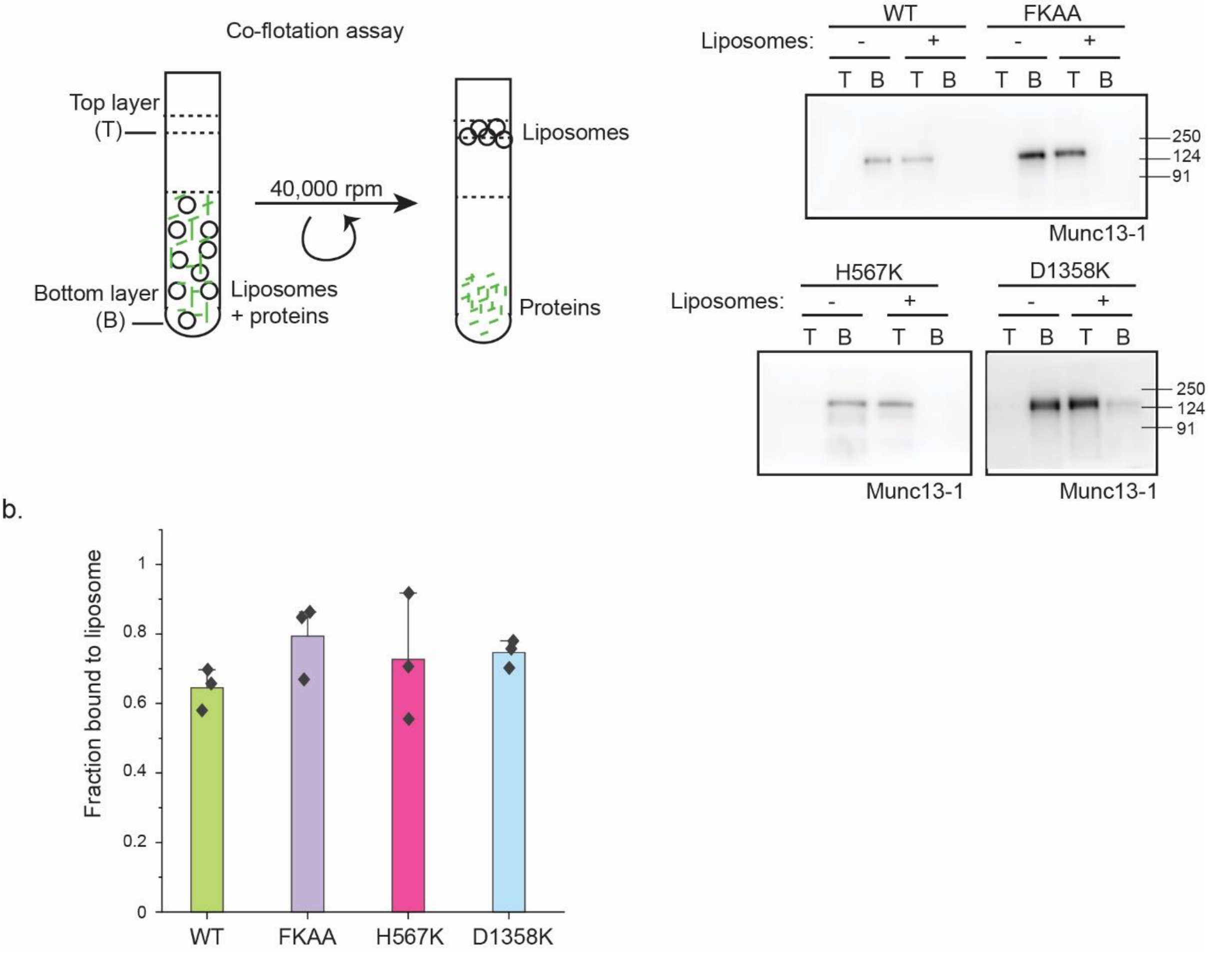

Fig. S6: Munc13-1 WT and its mutated variants interact with membrane lipids.

a,b, Cartoon showing co-flotation assay (a, left) where protein interacting with liposomes will be in the top layer (T) and unbound proteins would be in bottom layer (B). Representative immunoblots (a, right) and the quantification (b) of co-flotation assay showing the interaction of WT and mutants of Munc13-1 (as indicated) with liposomes; n=3 independent experiments. Molecular weights (in kDa) are shown. Antibody used has been mentioned. Data represented as mean±SEM.

Fig. S7

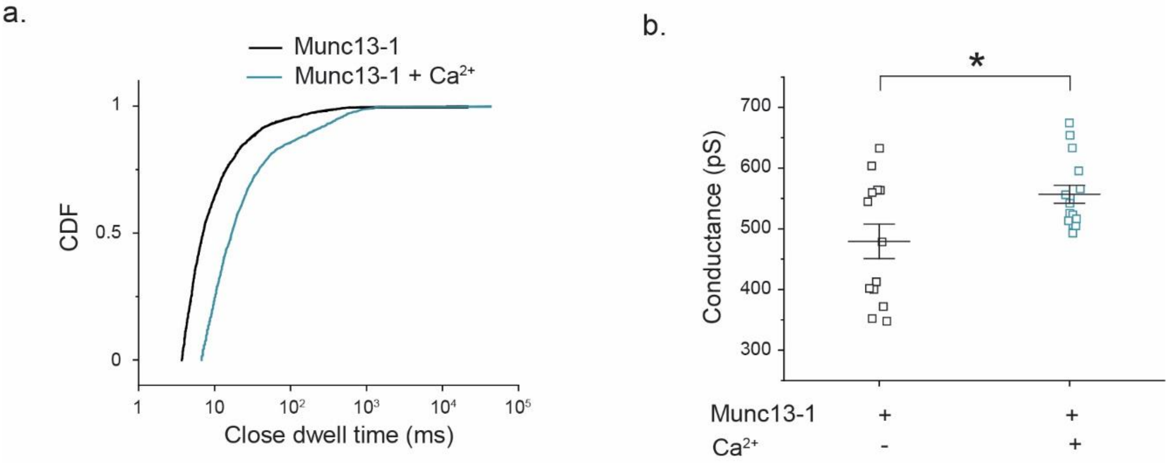

Fig. S7: Effect of Ca2+ in Munc13-1 triggered pores.

a, Cumulative distribution functions (CDF) of closed dwell times for each experimental condition as indicated. n = 3 independent BLM recordings, for (Munc13-1 + Ca^{2+}) condition. **b,** Scatter plots show comparison of pore conductance between apo-/Munc13-1 (black) and Ca^{2+}/Munc13-1 (blue) triggered pores, as indicated; n=13 (for Munc13-1), n=15 (for Munc13-1+ Ca^{2+}) single pores. Students T test was performed to compare the two means; *p<0.05. Data represented as mean±SEM

Fig. S8

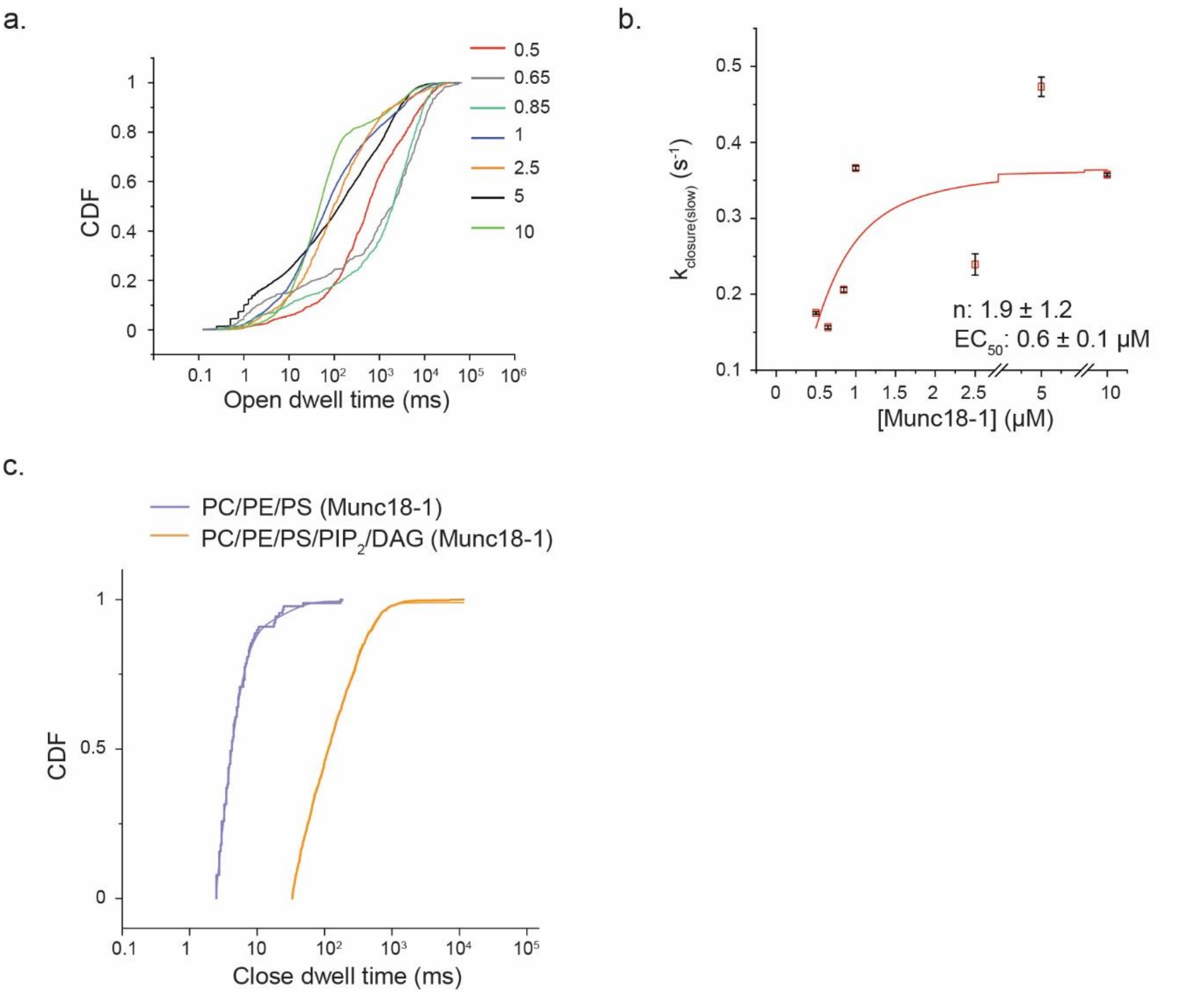

Fig. S8: Munc18-1 kinetically alters fusion pore properties.

a, Cumulative distribution functions (CDF) of open dwell times for indicated Munc18-1 concentrations. n = 3 independent BLMs. Two to three sets of NDs were used. **b,** The open state CDFs from **a,** were fitted with three exponential equations and the slowest kinetic rate constants ($k_{closure(slow)}$) are plotted as a function of [Munc18-1]. The data was fitted with a Hill equation and the derived parameters n (Hill coefficient) and EC_{50} are shown. **c,** Cumulative distribution functions (CDF) of close dwell times for indicated BLM lipids, in the presence of Munc18-1. Data represented as mean ± SEM. n = 3 independent BLMs; two to three sets of NDs were used.

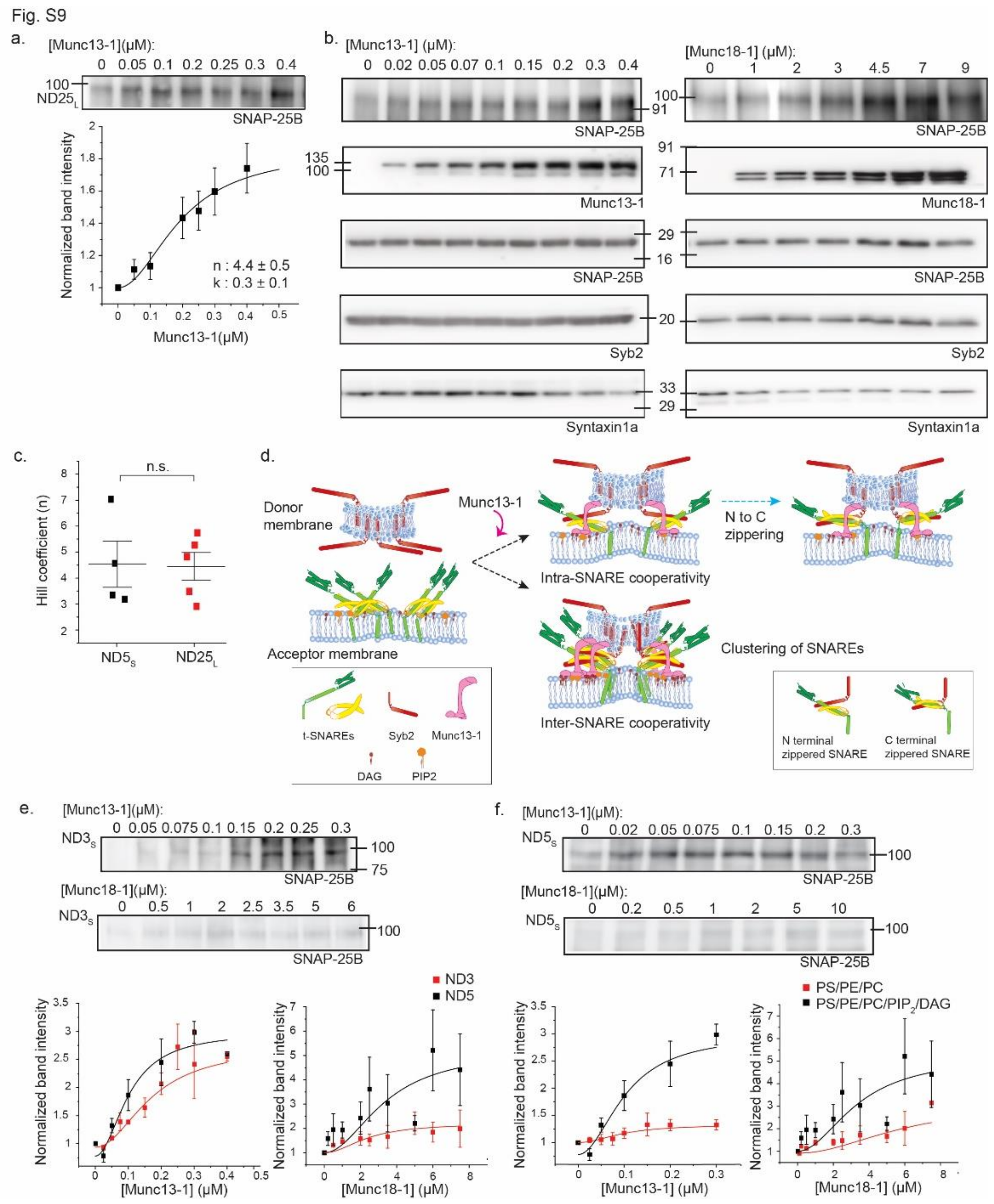

Fig. S9: Munc13-1 and Munc18-1 enhance trans-SNARE assembly.

a, Representative immunoblot showing trans SNARE complexes during t-SNARE liposomes' fusion with $ND25_L$ with increasing [Munc13-1] (top). Scatter plots fitted with modified hill equation showing fold change in band intensity for trans-SNARE complexes formed as [Munc13-1] (bottom) increases. The hill coefficient (n) and EC_{50} (k) are indicated in the plot. The membrane lipid composition for the t-SNARE liposomes was PS/PE/PC/PIP_2/DAG. **b,** Representative immunoblots showing monomeric SNAREs originating from the heated trans SNARE complexes formed in presence of Munc13-1 (left) and Munc18-1 (right). **c,** Scatter plot shows pooled data for hill coefficients in presence of Munc13-1 for $ND5_S$(black), and $ND25_L$(red); n = 5 (for $ND25_L$) independent trials. **d,** Illustration shows two modes of

cooperative SNARE complex organization by Munc13-1. Intra-SNARE cooperativity indicates cooperative zippering of trans-SNARE complexes from N- to C-terminus. Inter-SNARE cooperativity indicates cooperative clustering of multiple SNARE complexes at the release site. **e,** Representative immunoblot showing trans SNARE complexes during t-SNARE liposomes' fusion with $ND3_S$ with increasing [Munc13-1] (top) and [Munc18-1] (bottom). Scatter plots fitted with modified hill equation showing fold change in band intensity for trans-SNARE complexes formed as [Munc13-1] (bottom, left) and [Munc18-1] (bottom, right) increases for $ND3_S$ and $ND5_S$; n=4 (for $ND3_S$/Munc13-1), n=3 (for $ND3_S$/Munc18-1), independent blots were analysed. The membrane lipid composition for the t-SNARE liposomes was PS/PE/PC/PIP2/DAG. **f,** Representative immunoblot showing trans SNARE complexes during t-SNARE liposomes' fusion with $ND5_S$ with increasing [Munc13-1] (top) and [Munc18-1] (bottom), using membrane lipid composition: PS/PE/PC; n=3 (for $ND5_S$/Munc13-1) and n=4 (for $ND5_S$/Munc18-1) independent blots were analysed. Scatter plots fitted as in (e) for increasing [Munc13-1] (bottom, left) and [Munc18-1] (bottom, right) for different membrane lipid compositions as indicated. Molecular weights (in kDa) are shown. Data represented as mean±SEM.

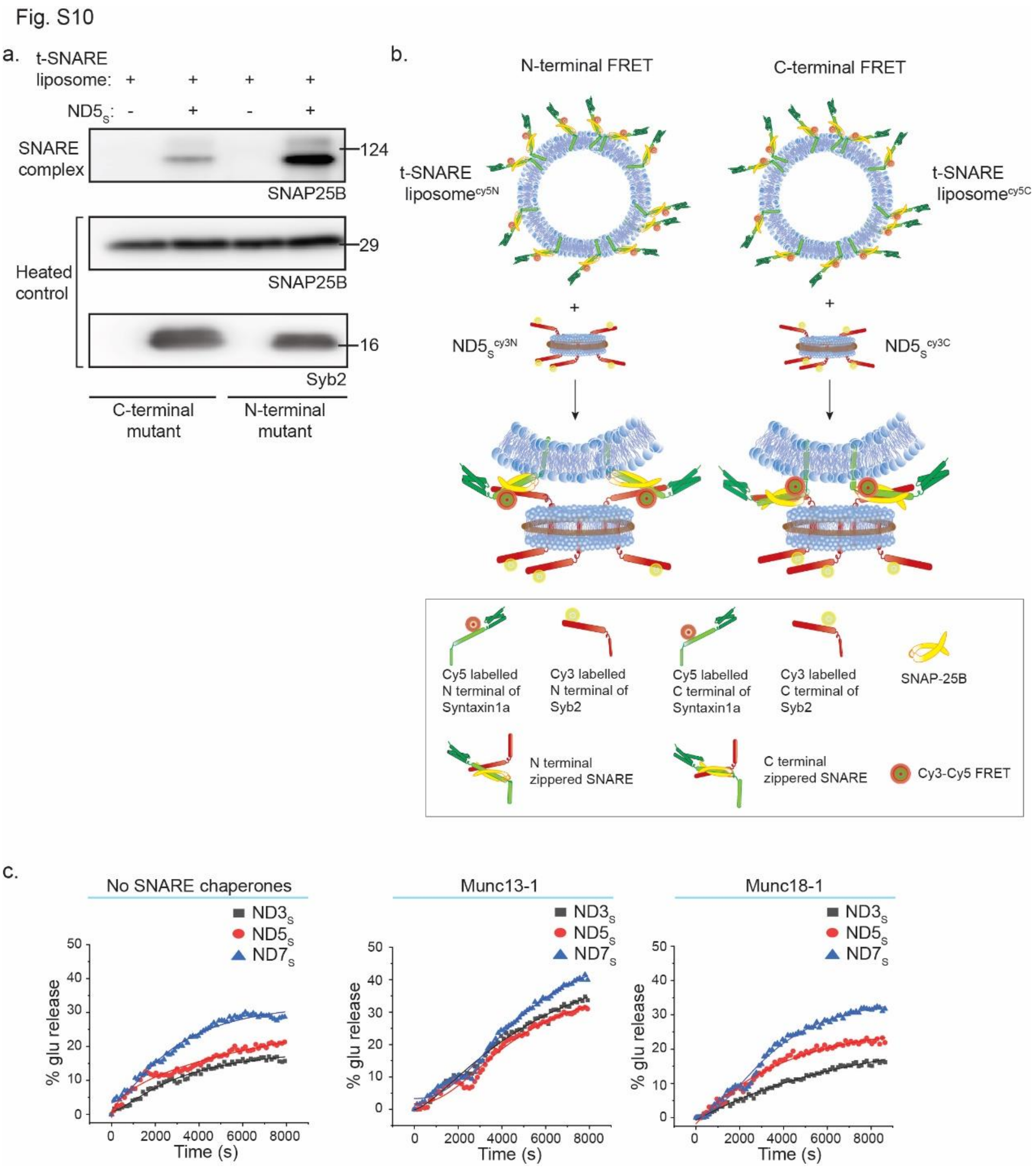

Fig. S10: Munc13-1 and Munc18-1 enhance kinetics of SNARE zippering.

a, Immunoblot show trans-SNARE complex formation by the following ND5 and t-SNARE liposome combinations – 1) $ND5_S$ containing syb2 (Q33C) reacts with t-SNARE liposome containing syntaxin-1a (I203C); 2) $ND5_S$ containing syb2 (A72C) reacts with t-SNARE liposome containing syntaxin-1a (V241C). Molecular weights (in kDa) are shown. **b,** Illustration shows FRET assay performed with ND and t-SNARE liposome pairs as indicated. **c,** Representative time course of glutamate release through the fusion pores during fusion reaction as $ND3_S$ (black), $ND5_S$ (red) and $ND7_S$ (blue) react with glutamate entrapped t-SNARE liposomes, in separate set of experiments, in the absence (left) and presence of SNARE chaperones Munc13-1 (middle) and Munc18-1 (right).

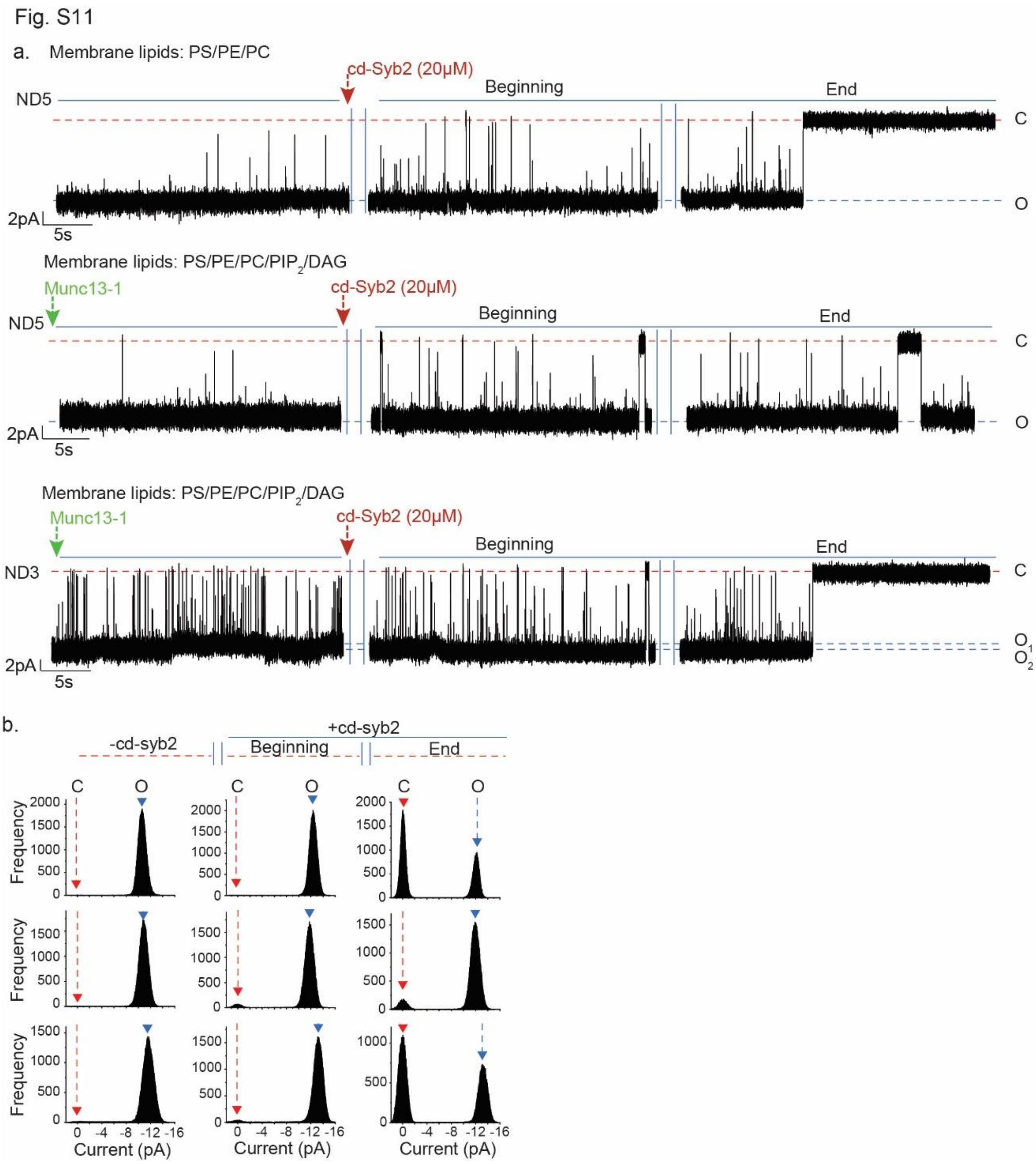

Fig. S11: cd-syb2 titration of trans-SNARE complexes in the presence of Munc13-1.

a-c, Representative traces of $ND5_S$ and $ND3_S$ pores, in absence and presence of Munc13-1, membrane lipid composition are mentioned. The addition of different [cd-syb2] is indicated by the arrow. The corresponding current histograms from traces shown in (a) are plotted. The close (C) and open (O) states are indicated. n = 3 independent BLM recordings.

Fig. S12

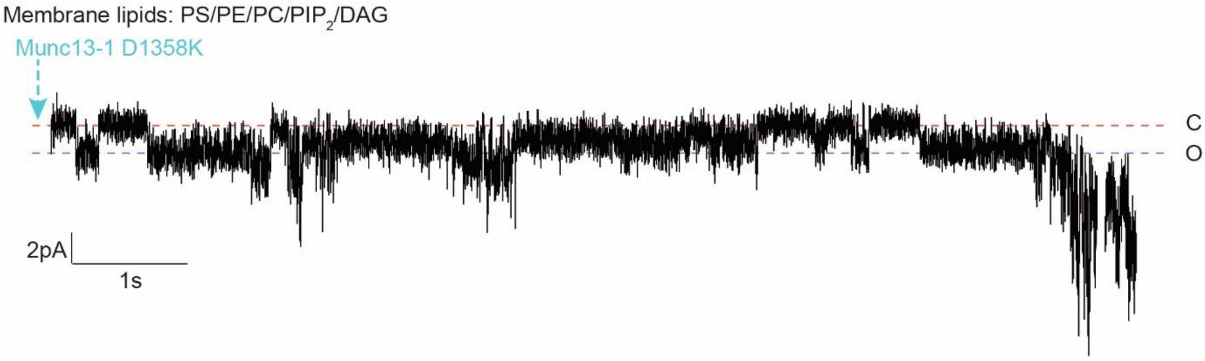

Fig. S12: Phenotype of Munc13-1(D1358K) triggered ND5$_S$ pores.

Representative trace of ND5$_S$ pores where Munc13-1(D1358K) was added at the beginning of pore opening. The close (c) and open (o) states are indicated. n = 3 independent BLM recordings.

Tables

Table S1: Time constants derived from exponential fits of CDFs for ND5$_S$ pores' closed dwell times under indicated conditions.

BLM lipids	T1 (ms)	T2 (ms)	T3 (ms)
PS/PE/PC	1.08022 ± 0.0929	11.20903 ± 0.59279	
PS/PE/PC/PIP$_2$/DAG	47.63102 ± 0.60803	433.95019 ± 5.13404	3652.7983 ± 29.69766
PS/PE/PC/PIP$_2$	1.59403 ± 0.03398	8.47616 ± 1.12514	73.75739 ± 7.17722

Table S2: Time constants derived from exponential fits of CDFs for $ND5_S$ pores' closed dwell times under indicated conditions.

SNARE chaperones	BLM lipids	T1 (ms)	T2 (ms)	T3 (ms)
Munc13-1 (all conductance)	PS/PE/PC/PIP_2/DAG	2.17374 ± 0.01337	10.85632 ± 0.15845	107.42656 ± 4.71692
Munc13-1 (O1 conductance state)	PS/PE/PC/PIP_2/DAG	1.63832 ± 0.07127	13.11495 ± 0.45057	172.77384 ± 5.60097
Munc13-1 (O2 conductance state)	PS/PE/PC/PIP_2/DAG	1.88948 ± 0.02756	7.54499 ± 0.42755	29.38068 ± 2.74193
Munc13-1 (H567K)	PS/PE/PC/PIP_2/DAG	19.088 ± 1.0916	86.83327 ± 2.72137	1282.801 ± 82.77855
Munc13-1 (FKAA)	PS/PE/PC/PIP_2/DAG	14.04542 ± 0.78951	44.26998 ± 2.68866	287.91322 ± 18.36594
Munc18-1 (1uM)	PS/PE/PC/PIP_2/DAG	11.23319 ± 0.39066	59.28863 ± 0.49511	6784.61293 ± 1398.82225
Munc18-1 (D326K) (1uM)	PS/PE/PC/PIP2/DAG	1.69582 ± 0.04004	19.8069 ± 0.98981	269.22648 ± 36.18089
Munc18-1 (5 uM)	PS/PE/PC	2.14756 ± 0.07453	21.37497 ± 7.73946	
Munc18-1 (5 uM)	PS/PE/PC/PIP_2/DAG	8.8877 ± 0.45884	44.29575 ± 1.11973	231.43921 ± 1.65097
Munc13-1 addition to Munc18-1 pore	PS/PE/PC/PIP_2/DAG	1.07043 ± 0.02343	4.3718 ± 0.50025	69.20933 ± 11.90592
Munc18-1 addition to Munc13-1 pore	PS/PE/PC/PIP_2/DAG	1.0354 ± 0.0713	6.78383 ± 0.30642	116.91332 ± 17.95667
Munc13-1 and Munc18-1	PS/PE/PC/PIP_2/DAG	1.78341 ± 0.04036	9.32968 ± 0.31637	

Table S3: Time constants derived from exponential fits of CDFs for $ND5_S$ pores' open dwell times at different [Munc18-1].

[Munc18-1] (μM)	T1 (ms)	T2 (ms)	T3 (ms)
0.5	8.3914 ± 0.49275	420.96642 ± 1.89881	5698.62763 ± 50.60778
0.65	2.72765 ± 0.12757	467.1525 ± 21.32151	6387.06078 ± 80.36214
0.85	11.76209 ± 0.38738	1012.36185 ± 68.10406	4855.73469 ± 71.1979
1	19.29365 ± 0.13299	127.39351 ± 0.92612	2731.51159 ± 23.45176
2.5	42.69668 ± 0.64331	311.21785 ± 7.24682	4179.57244 ± 244.81788
5	1.00825 ± 0.00934	384.00959 ± 46.56108	2112.3612 ± 57.0785
10	53.92856 ± 0.34812	22.74097 ± 0.7809	2798.03494 ± 16.32121

Table S4: List of Antibodies used.

Antibody (Ab)	Catalogue No	Company	Dilution
SNAP-25 Rabbit mAb	5309S	Cell Signaling Technology (Danvers, MA)	1:3000 to 1:5000
VAMP2 Rabbit mAb	13508S	Cell Signaling Technology (Danvers, MA)	1:5000
Munc18-1Rabbit pAb	ab3451	abcam	1:5000 (For WB) 1:500 (For ICC)
Munc13-1Shp pAb	AB215426	abcam	1:2000
Anti-rabbit IgG, HRP-linked	7074S	Cell Signaling Technology (Danvers, MA)	1:3000 to 1:5000
Rabbit Anti-Sheep IgG H&L (HRP)	ab97130	abcam	1:2000
Munc13-1 Rabbit pAb	126 103	Synaptic Systems	1:500
SNAP25 Mouse mAb	MA5-17609	Invitrogen	1:200
Anti-mouse IgG Fab2 Alexa Fluor(R) 555 Molecular Probes	4409S	Cell Signaling Technology (Danvers, MA)	1:1000

Anti-rabbit IgG Fab2 Alexa Fluor(R) 488 Molecular Probes	4412S	Cell Signaling Technology (Danvers, MA)	1:1000
Goat anti-Guinea Pig IgG (H+L) Highly Cross-Adsorbed Secondary Antibody, Alexa Fluor™ 647	A-21450	ThermoFisher Scientific	1:1000
Synaptobrevin 2 Mouse mAb	104211	Synaptic Systems	1:500
Syntaxin 1 Mouse mAb	110011	Synaptic Systems	1:500
Synapsin Guinnea pig mAb	106308	Synaptic Systems	1:1000

Table S5: List of primers used for In-Fusion cloning.

Primers	Sequence
Munc13-1(H567K) Forward	CACGCCGAAGAACTTCGAGGTGTGGACGGC
Munc13-1(H567K) Reverse	AAGTTCTTCGGCGTGGTGCAGGAGATGG
Munc13-1(FKAA) Forward	GCGCGCCGCCGCCACCATTAGCAATGTGCTTCTCC
Munc13-1(FKAA) Reverse	GTGGCGGCGGCGCGCCGCATGTAGTGGCC
Munc13-1(D1358K) Forward	GGACGCAAAGAACGTGCTACAGCCCATCATGG
Munc13-1(D1358K) Reverse	ACGTTCTTTGCGTCCTGTGCCACGCTGC
Munc18-1(D326K) Forward	CATGCGGAAGCTGTCCCAGATGCTGAAGAAAATGC
Munc18-1(D326K) Reverse	GACAGCTTCCGCATGGTGGTCTTCTCTCC
