# Supplementary material for: Differential SNARE chaperoning by Munc13-1 and Munc18-1 dictates fusion pore fate at the release site
Source: Nat Commun. 2024 May 16;15:4132. doi: 10.1038/s41467-024-46965-7 (PMC11099066; doi:10.1038/s41467-024-46965-7)
Supplement: Supplementary file 3 — Reporting Summary [file 41467_2024_46965_MOESM3_ESM.pdf]

nature portfolio

Corresponding author(s): Debasis Das

Last updated by author(s): Mar 1, 2024

Reporting Summary

Nature Portfolio wishes to improve the reproducibility of the work that we publish. This form provides structure for consistency and transparency in reporting. For further information on Nature Portfolio policies, see our Editorial Policies and the Editorial Policy Checklist.

Statistics

For all statistical analyses, confirm that the following items are present in the figure legend, table legend, main text, or Methods section.

n/a	Confirmed	
☐	☒	The exact sample size (*n*) for each experimental group/condition, given as a discrete number and unit of measurement
☐	☒	A statement on whether measurements were taken from distinct samples or whether the same sample was measured repeatedly
☐	☒	The statistical test(s) used AND whether they are one- or two-sided<br>*Only common tests should be described solely by name; describe more complex techniques in the Methods section.*
☒	☐	A description of all covariates tested
☒	☐	A description of any assumptions or corrections, such as tests of normality and adjustment for multiple comparisons
☐	☒	A full description of the statistical parameters including central tendency (e.g. means) or other basic estimates (e.g. regression coefficient) AND variation (e.g. standard deviation) or associated estimates of uncertainty (e.g. confidence intervals)
☐	☒	For null hypothesis testing, the test statistic (e.g. *F*, *t*, *r*) with confidence intervals, effect sizes, degrees of freedom and *P* value noted<br>*Give P values as exact values whenever suitable.*
☒	☐	For Bayesian analysis, information on the choice of priors and Markov chain Monte Carlo settings
☒	☐	For hierarchical and complex designs, identification of the appropriate level for tests and full reporting of outcomes
☒	☐	Estimates of effect sizes (e.g. Cohen's *d*, Pearson's *r*), indicating how they were calculated

Our web collection on statistics for biologists contains articles on many of the points above.

Software and code

Policy information about availability of computer code

Data collection	pClamp 10 software for single channel BLM electrophysiology data collection<br>Amersham imager 600 for western blot imaging<br>Olympus FV3000 confocal microscope for fluorescence imaging<br>Cytiva AKTApure and Unicorn 6 for gel filteration and peak measurement<br>Spark control Magellan v3.1std for fluorescence reading in TECAN<br>Fiji (ImageJ) for western blot band intensity measurement<br>Adobe Illustrator 2023 for schematics and cartoon model
Data analysis	Image analysis: Fiji (ImageJ)<br>Data analysis and graph plotting: Microsoft excel, Origin 2020b<br>Single channel BLM recording analysis: Clampfit 10.7.0

For manuscripts utilizing custom algorithms or software that are central to the research but not yet described in published literature, software must be made available to editors and reviewers. We strongly encourage code deposition in a community repository (e.g. GitHub). See the Nature Portfolio guidelines for submitting code & software for further information.

Data

Policy information about availability of data

All manuscripts must include a data availability statement. This statement should provide the following information, where applicable:

- Accession codes, unique identifiers, or web links for publicly available datasets
- A description of any restrictions on data availability
- For clinical datasets or third party data, please ensure that the statement adheres to our policy

All data necessary to support and validate the experiments have been provided in the Supplementary Information file as well as Source data file. The representative raw single pore traces and the quantified parameters from each individual trials are already provided in the manuscript. Still, all the raw single pore traces are available to the reader upon request.

Research involving human participants, their data, or biological material

Policy information about studies with human participants or human data. See also policy information about sex, gender (identity/presentation), and sexual orientation and race, ethnicity and racism.

Reporting on sex and gender	Not applicable
Reporting on race, ethnicity, or other socially relevant groupings	Not applicable
Population characteristics	Not applicable
Recruitment	Not applicable
Ethics oversight	Not applicable

Note that full information on the approval of the study protocol must also be provided in the manuscript.

Field-specific reporting

Please select the one below that is the best fit for your research. If you are not sure, read the appropriate sections before making your selection.

- [x] Life sciences
- [ ] Behavioural & social sciences
- [ ] Ecological, evolutionary & environmental sciences

For a reference copy of the document with all sections, see nature.com/documents/nr-reporting-summary-flat.pdf

Life sciences study design

All studies must disclose on these points even when the disclosure is negative.

Sample size	Sample sizes were determined based on variabiliy in the data from initial pilot studies. No statistical methods were used to calculate sample size. Sample sizes used sufficiently indicated consistent trend in the results obtained.
Data exclusions	No data were excluded.
Replication	Each experiments were performed multiple times for data reproducibility as mentioned in figure legends of the respective data. Materials for in-vitro experiments were freshly prepared for experiments atleast thrice.
Randomization	This study did not incorporate a formal randomization process as it was not relevant for our in-vitro experiments. Neuronal cell cultures plates were randomly picked for control and PMA treatment from cortices dissected from same litter. Samples like nanodiscs, liposomes and proteins prepared in each batch were randomly distributed for experimental conditions.
Blinding	Investigators were blinded to group allocation during data collection and analysis.

Reporting for specific materials, systems and methods

We require information from authors about some types of materials, experimental systems and methods used in many studies. Here, indicate whether each material, system or method listed is relevant to your study. If you are not sure if a list item applies to your research, read the appropriate section before selecting a response.

Materials & experimental systems

n/a	Involved in the study
	☒ Antibodies
☒	☐ Eukaryotic cell lines
☒	☐ Palaeontology and archaeology
	☒ Animals and other organisms
☒	☐ Clinical data
☒	☐ Dual use research of concern
☒	☐ Plants

Methods

n/a	Involved in the study
☒	☐ ChIP-seq
☒	☐ Flow cytometry
☒	☐ MRI-based neuroimaging

Antibodies

Antibodies used

1. Anti-SNAP-25 [Cell Signaling technology, 5309, Clone D9A12, Lot 1]
2. Anti-VAMP2 [Cell Signaling technology, 13508, Clone D6O1A, Lot 1]
3. Anti-Munc18-1 [Abcam, ab3451, Lot GR3261450-3]
4. Anti-Munc13-1 [Abcam, ab215426, Lot 1024977-1]
5. Anti-Munc13-1[Synaptic systems, 126103]
6. Goat Anti-rabbit IgG-HRP [Cell Signaling technology, 7074, Lot 28]
7. Rabbit Anti-Sheep IgG H&L (HRP) [abcam, ab97130, Lot GR3312797-2]
8. Anti-SNAP-25[Invitrogen, MA5-17609, Clone SP12, Lot WG3336082]
9. Goat Anti-mouse IgG (H+L) F(ab')2 Alexa Fluor 555 [Cell Signaling technology, 4409, Lot 18]
10. Goat Anti-rabbit IgG (H+L) F(ab')2 Fragment Alexa Fluor 488 [Cell Signaling technology, 4412, Lot 22]
11. Goat anti-Guinea Pig IgG (H+L) Alexa Fluor 647 [ThermoFischer Scientific, A-21450, RRID AB_2735091, Lot 2633525]
12. Anti-Synaptobrevin2 [Synaptic systems, 104211, Clone 69.1]
13. Anti-Syntaxin1 [Synaptic systems, 110011, Clone 78.2]
14. Anti-Synapsin1 [Synaptic systems, 106308, Clone Gp46.1]

Validation

The antibody list provided in the supplementary file contains names of the companies from where they were procured, with the catalogue numbers. The respective company websites contain the relevant validation statement.

Anti-SNAP-25 [Cell Signaling technology, 5309]: Suitable for WB, IP, IHC; Reacts with Human, Mouse, Rat
https://www.cellsignal.com/products/primary-antibodies/snap25-d9a12-rabbit-mab/5309

Anti-VAMP2 [Cell Signaling technology, 13508]: Suitable for WB, IP, IHC; Reacts with Human, Mouse, Rat
https://www.cellsignal.com/products/primary-antibodies/vamp2-d6o1a-rabbit-mab/13508

Anti-Munc18-1 [Abcam, ab3451]: Suitable for WB, ICC; Reacts with Human, Mouse, Rat
https://www.abcam.com/en-in/products/primary-antibodies/munc18-1-antibody-ab3451#

Anti-Munc13-1 [Abcam, ab215426]: Suitable for WB; Reacts with Mouse
https://www.abcam.com/en-in/products/primary-antibodies/munc13-1-antibody-c-terminal-ab215426#

Anti-Munc13-1[Synaptic systems, 126103]: Suitable for WB, IP, IHC, ICC; Reacts with Human, Mouse, Rat
https://sysy.com/product/126103

Goat Anti-rabbit IgG-HRP [Cell Signaling technology, 7074]: Suitable for WB; Reacts with Rabbit
https://www.cellsignal.com/products/secondary-antibodies/anti-rabbit-igg-hrp-linked-antibody/7074

Rabbit Anti-Sheep IgG H&L (HRP) [abcam, ab97130]: Suitable for WB, IHC, ICC; Reacts with Sheep
https://www.abcam.com/en-in/products/secondary-antibodies/rabbit-sheep-igg-h-l-hrp-ab97130

Anti-SNAP-25[Invitrogen, MA5-17609]: Suitable for WB, ICC; Reacts with Human, Mouse, Rat
https://www.thermofisher.com/antibody/product/SNAP25-Antibody-clone-SP12-Monoclonal/MA5-17609

Goat Anti-mouse IgG (H+L) F(ab')2 Alexa Fluor 555 [Cell Signaling technology, 4409]: Suitable for ICC; Reacts with Mouse
https://www.cellsignal.com/datasheet.jsp?productId=4409&images=1&size=A4

Goat Anti-rabbit IgG (H+L) F(ab')2 Alexa Fluor 488 [Cell Signaling technology, 4412]: Suitable for ICC; Reacts with rabbit
https://www.cellsignal.com/datasheet.jsp?productId=4412&images=1&size=A4

Goat anti-Guinea Pig IgG (H+L) Alexa Fluor 647 [ThermoFischer Scientific, A-21450], Suitable for WB, IHC, ICC; Reacts with Guinea Pig
https://www.thermofisher.com/antibody/product/Goat-anti-Guinea-Pig-IgG-H-L-Highly-Cross-Adsorbed-Secondary-Antibody-Polyclonal/A-21450

Anti-Synaptobrevin2 [Synaptic systems, 104211]: Suitable for WB, IP, IHC, ICC; Reacts with Human, Mouse, Rat, Hamster

https://sysy.com/product/104211

Anti-Syntaxin1 [Synaptic systems, 110011]: Suitable for WB, IP, IHC, ICC; Reacts with Human, Mouse, Rat
https://sysy.com/product/110011

Anti-Synapsin1 [Synaptic systems, 106308]: Suitable for WB, IP, IHC, ICC; Reacts with Human, Mouse, Rat
https://sysy.com/product/106308

Animals and other research organisms

Policy information about studies involving animals; ARRIVE guidelines recommended for reporting animal research, and Sex and Gender in Research

Laboratory animals	P0-P4 Sprague-Dawley rats
Wild animals	this study did not involve wild animals
Reporting on sex	this information was not collected, as our study does not report sex specific alteration of results.
Field-collected samples	this study did not involve samples collected from field.
Ethics oversight	Experimental procedures described below were in accordance with the guidelines of the Committee for Supervision and Care of Experimental Animals (CPCSEA), Government of India, and were approved by the TIFR Institutional Animal Ethics committee (TIFR/IAEC/2020-4).

Note that full information on the approval of the study protocol must also be provided in the manuscript.

Plants

Seed stocks	Not applicable for the current study
Novel plant genotypes	Not applicable for the current study
Authentication	Not applicable for the current study
